# Supplementary material for: Poor air quality is associated with impaired visual cognition in the first two years of life: A longitudinal investigation
Source: eLife. 2023 Apr 25;12:e83876. doi: 10.7554/eLife.83876 (PMC10129323; doi:10.7554/eLife.83876)

**Supplementary File 1**. Correlation table showing pairwise correlations for the key measures from the present study. AQI = air quality index; SES = SES score from the Kuppuswamy scale; PropC = ‘first-look change’ change preference score; PropNC = ‘first-look no-change’ change preference score; SR = shift rate. First index number indicates load (1 = Low, 2 = Medium, 3 = High) and second index number indicates year (1 or 2). Colors reflect the strength of the correlation (see bar).


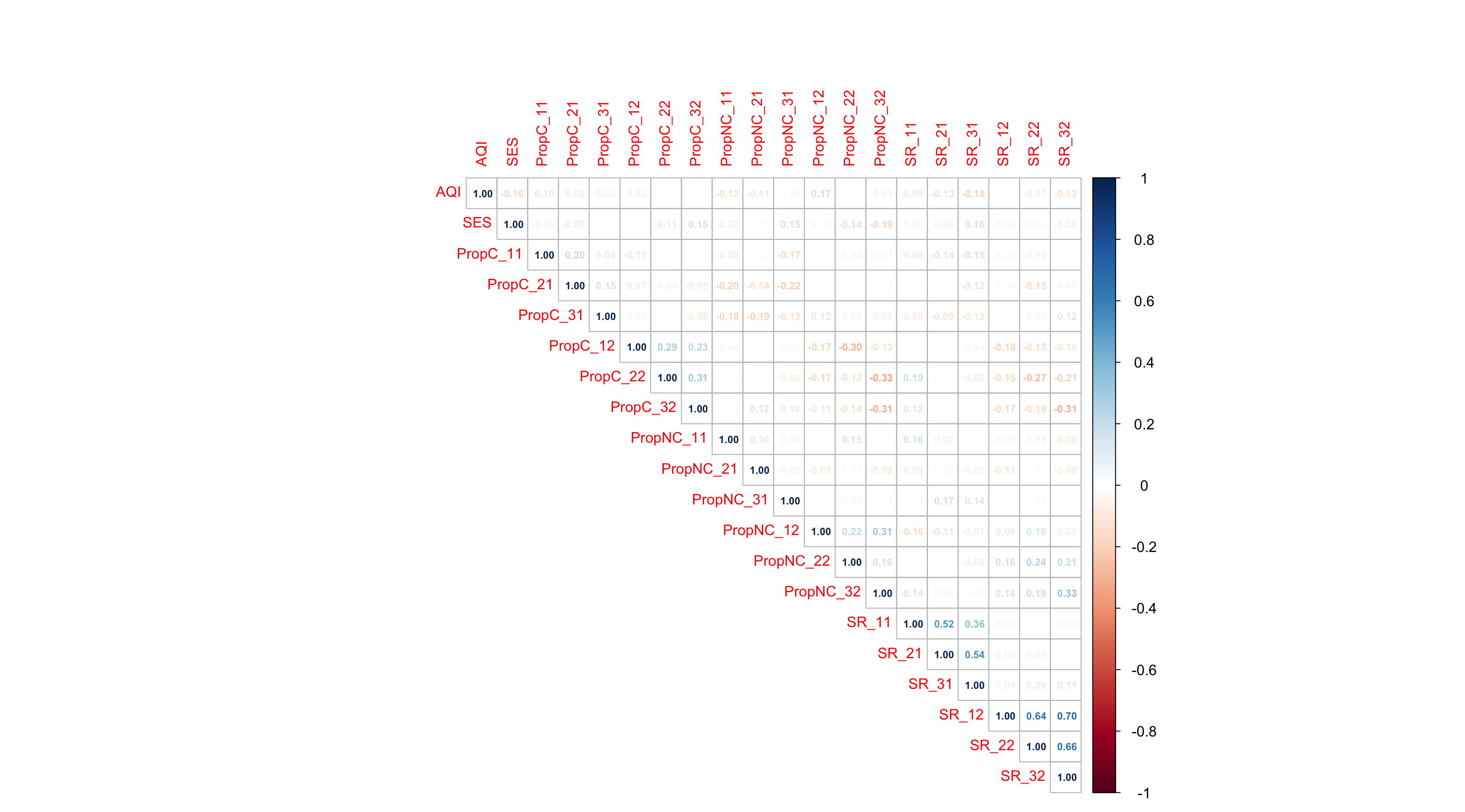

Supplement: Supplementary file 1. — AQI = air quality index; SES = SES score from the Kuppuswamy scale; PropC = ‘first-look change’ change preference score; PropNC = ‘first-look no-change’ change preference score; SR = shift rate. First index number indicates load (1=Low, 2=Medium, 3=High) and second index number indicates year (1 or 2). Colors reflect the strength of the correlation (see bar). [file elife-83876-supp1.docx]
