# Supplementary material for: Poor air quality is associated with impaired visual cognition in the first two years of life: A longitudinal investigation
Source: eLife. 2023 Apr 25;12:e83876. doi: 10.7554/eLife.83876 (PMC10129323; doi:10.7554/eLife.83876)
Supplement: Supplementary file 2. — Model parameters for linear mixed-effect model assessing the impact of air quality (AQI) on the baseline change preference score model which included Year, Load, SES, Age Cohort, and LookingWindow1 as predictors, controlling for the shift rate (see Table 6). [file elife-83876-supp2.docx]

**Supplementary File 2.**

Model parameters for linear mixed-effect model assessing the impact of air quality (AQI) on the baseline change preference score model which included Year, Load, SES, Age Cohort, and LookingWindow1 as predictors, controlling for the shift rate (see Table 6).

| **Variable** | **Estimate** | **Std. Error** | **DF** | **t value** | **Pr(>\|t\|)** |
| --- | --- | --- | --- | --- | --- |
| (Intercept) | 0.376 | 0.016 | 646.80 | 23.364 | <0.001 |
| **SR** | **0.094** | **0.023** | **855.40** | **4.166** | **<0.001** |
| Year | -0.010 | 0.108 | 707.20 | -0.096 | 0.9232 |
| **Load1** | **0.026** | **0.009** | **834.30** | **2.821** | **0.0049** |
| Load2 | -0.004 | 0.009 | 829.60 | -0.404 | 0.6866 |
| SES | -0.011 | 0.014 | 538.00 | -0.796 | 0.4264 |
| Age | 0.027 | 0.014 | 185.70 | 1.835 | 0.068 |
| AQI | 0.000 | 0.000 | 181.30 | -0.547 | 0.5851 |
| Year:SES | 0.028 | 0.027 | 693.50 | 1.027 | 0.305 |
| Year:LookingWindow1 | 0.042 | 0.130 | 696.90 | 0.321 | 0.7483 |
| SES:LookingWindow1 | 0.012 | 0.017 | 543.90 | 0.724 | 0.4691 |
| **Year:AQI** | **0.001** | **0.000** | **916.50** | **2.011** | **0.0446** |
| Year:SES:LookingWindow1 | -0.043 | 0.033 | 689.20 | -1.287 | 0.1986 |
