# Supplementary material for: Poor air quality is associated with impaired visual cognition in the first two years of life: A longitudinal investigation
Source: eLife. 2023 Apr 25;12:e83876. doi: 10.7554/eLife.83876 (PMC10129323; doi:10.7554/eLife.83876)
Supplement: Supplementary file 3. — Model parameters for linear mixed-effect model assessing the impact of air quality (AQI) on the baseline visual processing speed (shift rate) model which included Year, Load, SES, and Age Cohort as predictors, controlling for the change preference score (see Table 7). [file elife-83876-supp3.docx]

**Supplementary File 3.**

Model parameters for linear mixed-effect model assessing the impact of air quality (AQI) on the baseline visual processing speed (shift rate) model which included Year, Load, SES, and Age Cohort as predictors, controlling for the change preference score (see Table 7)

| **Variable** | **Estimate** | **Std. Error** | **DF** | **t value** | **Pr(>\|t\|)** |
| --- | --- | --- | --- | --- | --- |
| (Intercept) | 0.548 | 0.022 | 742.80 | 24.621 | <0.001 |
| **Change Pref** | **0.193** | **0.040** | **936.40** | **4.798** | **<0.001** |
| Year | 0.011 | 0.017 | 892.40 | 0.669 | 0.50337 |
| **Load1** | **0.034** | **0.011** | **814.20** | **3.013** | **0.00267** |
| Load2 | 0.001 | 0.011 | 811.20 | 0.098 | 0.92203 |
| SES | 0.001 | 0.003 | 205.60 | 0.318 | 0.75072 |
| Age | 0.003 | 0.028 | 196.50 | 0.119 | 0.90528 |
| **AQI** | **-0.001** | **0.000** | **192.70** | **-2.067** | **0.04011** |
| Year:SES | -0.005 | 0.004 | 927.10 | -1.120 | 0.26281 |
