# Supplementary material for: Poor air quality is associated with impaired visual cognition in the first two years of life: A longitudinal investigation
Source: eLife. 2023 Apr 25;12:e83876. doi: 10.7554/eLife.83876 (PMC10129323; doi:10.7554/eLife.83876)
Supplement: Supplementary file 4. — Parameters include year, SES, Looking window 1 and age cohort. [file elife-83876-supp4.docx]

**Supplementary File 4**

Model parameters from linear model examining the effects of air quality on a change preference model in set size 2 only, across both years. Parameters include year, SES, Looking window 1 and age cohort.

| **Variable** | **Estimate** | **Std. Error** | **t value** | **Pr(>\|t\|)** |
| --- | --- | --- | --- | --- |
| (Intercept) | 0.447 | 0.090 | 4.975 | <0.001 |
| Year | -0.125 | 0.179 | -0.701 | 0.484 |
| SES | -0.001 | 0.003 | -0.328 | 0.743 |
| LookingWindow1 | 0.009 | 0.108 | 0.081 | 0.936 |
| Age | 0.040 | 0.022 | 1.771 | 0.077 |
| AQI | 0.000 | 0.000 | 0.285 | 0.776 |
| Year:SES | 0.003 | 0.043 | 0.059 | 0.953 |
| Year:LookingWindow1 | 0.223 | 0.215 | 1.036 | 0.301 |
| **Year:AQI** | **0.002** | **0.001** | **2.613** | **0.009** |
| Year:SES:LookingWindow1 | 0.006 | 0.053 | 0.107 | 0.915 |
