# Supplementary material for: Poor air quality is associated with impaired visual cognition in the first two years of life: A longitudinal investigation
Source: eLife. 2023 Apr 25;12:e83876. doi: 10.7554/eLife.83876 (PMC10129323; doi:10.7554/eLife.83876)
Supplement: Supplementary file 5. [file elife-83876-supp5.docx]

**Full set of assessments carried out in Project INDIA (I**nfant **N**eural and **D**yadic **I**nteraction **A**ssessment).

- Children’s VWM task (behaviour and brain function using fNIRS)
- Dyadic interactions between caregivers and children.
- Standardized assessments (Mullen in year 1 and ASQ in year 2)
- Anthropometry measurements
- Birth history questionnaire (year 1)
- SES/demographic assessment (year 1)
- Early language assessment (year 2)
- Caregiver’s VWM task (behaviour only in year 2)
- Domestic violence, depression, and empowerment interview (year 2)
- MRI scan in Lucknow (every 6 months)
- Home assessment during which the following were gathered:
  - 3-days of LENA recordings
  - 3-days of nutrition questionnaires
  - 3-days of sleep diaries
  - 3-days of fitbit recordings
  - Monitoring of in-home air quality
  - Anthropometry measurements
